# ENHANCING RESILIENCE IN OLDER ADULTS WITH ADVANCED LUNG CANCER

**DOI:** 10.1093/geroni/igac059.425

**Published:** 2022-12-20

**Authors:** Katherine Ramos

**Affiliations:** DUKE UNIVERSITY MEDICAL CENTER, Durham, North Carolina, United States

## Abstract

For older adults with advanced cancer, the negative impacts on their emotional and physical health are prevalent, often jeopardizing their capacity to effectively cope and exhibit resilience. Research on multidisciplinary interventions that promote resilience in older adults facing advanced lung cancer has received limited attention. To enhance how healthcare systems/teams may improve oncogeriatric care, we present study findings from a theory-driven behavioral intervention pilot (Self-System Therapy; SST) designed to target patient self-regulation (e.g., behavioral activation, promotion-focused goal setting) to enhance psychological and physical resilience. A total of 15 older adults with late-stage lung cancer completed 6–8-weeks of SST all delivered via videoconference. Self-report, accelerometry, and exit interview data will be presented along with prior pilot data of 30 older adults (total N = 45). Preliminary findings suggest that participants found the use of videoconference feasible and acceptable; with a 95% adherence rate, 7% attrition rate, and 95% satisfaction with SST.

